# Risk factors for early graft detachment requiring rebubbling in Descemet membrane endothelial keratoplasty with imported pre-cut donor tissues

**DOI:** 10.3389/fmed.2024.1266049

**Published:** 2024-02-08

**Authors:** Chung Young Kim, Chang Ho Yoon, Mee Kum Kim

**Affiliations:** ^1^Department of Ophthalmology, Seoul National University Hospital, Seoul, Republic of Korea; ^2^Department of Ophthalmology, Seoul National University College of Medicine, Seoul, Republic of Korea; ^3^Laboratory of Ocular Regenerative Medicine and Immunology, Artificial Eye Center, Seoul National University Hospital Biomedical Research Institute, Seoul, Republic of Korea; ^4^Transplantation Research Institute, Seoul National University Medical Research Center, Seoul, Republic of Korea

**Keywords:** Descemet membrane endothelial keratoplasty (DMEK), Fuchs endothelial dystrophy (FED), graft detachment, rebubbling, imported donor, intraocular pressure, diabetes

## Abstract

Graft detachment is a common and significant complication in Descemet membrane endothelial keratoplasty (DMEK). We investigated the risk factors of graft detachment requiring rebubbling after DMEK using imported pre-cut donor tissues. The medical records of 48 patients who underwent DMEK for Fuchs’ endothelial dystrophy (FED) or bullous keratopathy (BK) at Seoul National University Hospital were retrospectively reviewed. Donor, recipient, and surgical factors were evaluated using univariate and multivariate logistic regression models. Graft detachment requiring rebubbling occurred in 17 (32.7%) eyes. The detachment group exhibited older recipient age (*p* = 0.006), higher prevalence of diabetes (*p* = 0.001), and a higher proportion of FED (65%, *p* = 0.003). Notably, the detachment group demonstrated a significantly lower postoperative 2-h intraocular pressure (IOP) (*p* = 0.002) and a greater proportion of eyes with IOP <20 mmHg (*p* < 0.001). Older recipient age (OR 1.08, 95% CI 1.02–1.17), diabetes (OR 23.8, 95% CI 2.61–217), FED surgical indication (OR 6.19, 95% CI 1.74–22.0), lower postoperative 2-h IOP (OR 1.21, 95% CI 1.06–1.38), and postoperative 2-h IOP <20 mmHg (OR 14.0, 95% CI 1.64–119) were associated with increased odds of graft detachment. According to multivariate logistic regression, lower postoperative 2-h IOP (OR 1.23, 95% CI 1.02–1.47) and postoperative 2-h IOP <20 mmHg (OR 25.1, 95% CI 1.05–602) increased the risk of graft detachment. Lower postoperative 2-h IOP, particularly below 20 mmHg, may increase the risk of graft detachment, and diabetes in recipients may pose a higher risk of graft detachment after DMEK.

## Introduction

Since its introduction (1), Descemet membrane endothelial keratoplasty (DMEK) has emerged as the preferred treatment option for corneal endothelial disorders (2). Compared with conventional surgeries such as Descemet stripping automated endothelial keratoplasty, DMEK offers several advantages, including faster postoperative recovery, superior visual outcomes, and lower graft failure rates (3–5). Continuous improvements and technique standardization have further enhanced graft survival and surgical success (6, 7).

However, graft detachment remains a common and significant postoperative complication that concerns corneal surgeons. According to the American Academy of Ophthalmology Ophthalmic Technology Assessment, the incidence of graft detachment is as high as 28% (average ranging from 2 to 82%) (5). Graft detachment requires reattachment via air or sulfur hexafluoride (SF_6_) injection into the anterior chamber, known as rebubbling (8, 9). Importantly, graft detachment and the subsequent need for rebubbling are associated with increased endothelial cell loss and graft failure. Therefore, investigating risk factors of graft detachment is imperative to prevent graft failure.

Previous studies have proposed various strategies to minimize graft detachment, including donor selection based on age (10), modifications of descemetorrhexis size (11), intraocular pressure (IOP) adjustments (12), and SF_6_ injection instead of air (8). However, studies investigating the risk factors of graft detachment remain relatively limited. Moreover, the identified risk factors in existing studies show varying validity (11, 13–18). In addition, no reports have investigated the risk factors in DMEK using internationally imported pre-cut graft, which has a long preservation time inevitably.

In the current study, we conducted a comprehensive investigation of the donor, recipient, and surgical risk factors associated with early graft detachment requiring rebubbling after DMEK using pre-cut donor tissues. We aimed to compare and assess these factors in relation to previous reports, thus contributing to a better understanding of graft detachment risk factors in DMEK using internationally delivered pre-cut tissues.

## Materials and methods

### Study design and participants

This retrospective study was approved by the Seoul National University Institutional Review Board (IRB No.H-2304-005-1417) and adhered to the principles outlined in the Declaration of Helsinki. The requirement for informed consent was waived by the Institutional Review Board, considering the retrospective nature of the study.

This monocentric, interventional case series included 52 eyes of 48 patients who underwent DMEK for Fuchs’ endothelial dystrophy (FED) or bullous keratopathy (BK) between October 2016 and December 2022. Patients who were followed up shorter than 12 months were excluded. The medical records of all patients who underwent DMEK were reviewed. All patients were pseudophakic after either DMEK alone or in combination with cataract surgery. The surgical indication for DMEK was persistent corneal stromal edema accompanied by decreased visual acuity.

### Surgical procedure

The DMEK surgeries in this study were performed by a single surgeon (MKK) using a previously published (19) standardized technique. The donor grafts for DMEK were provided by the Eversight Eye Bank (Chicago, IL, United States) following standard procedures (20). Donor tissues with a death-to-operation interval of fewer than 7 days were utilized. Before DMEK, peripheral laser iridotomy or surgical iridectomy was performed during cataract surgery. The size of the recipient’s Descemet membrane stripping was typically 0.5–0.75 mm larger than that of the donor graft (7.5–8.5 mm). Grafts were inserted either endothelium-out using a Jones tube (Gunther Weiss Scientific, Hillsboro, OR, United States) or endothelium-in using a Coronet EndoGlide insertion device (Network Medical Products, Ripon, United Kingdom) after August 2018 owing to the commercial availability of EndoGlide in Korea. The graft was unfolded using tapping procedures and secured in place with an air tamponade (80–95%), as confirmed by the S mark. Patients received systemic steroid treatment and were prescribed topical 1% prednisolone acetate (Pred Forte; Allergan, Dublin, Ireland) and 0.5% moxifloxacin eye drops (Vigamox, Alcon, Geneva, Switzerland) administered four times daily. Follow-up visits were scheduled for 1 day, 2 days, 1 week, 1 month, 3 months, 6 months, and every 6 months thereafter.

**Figure 1:**
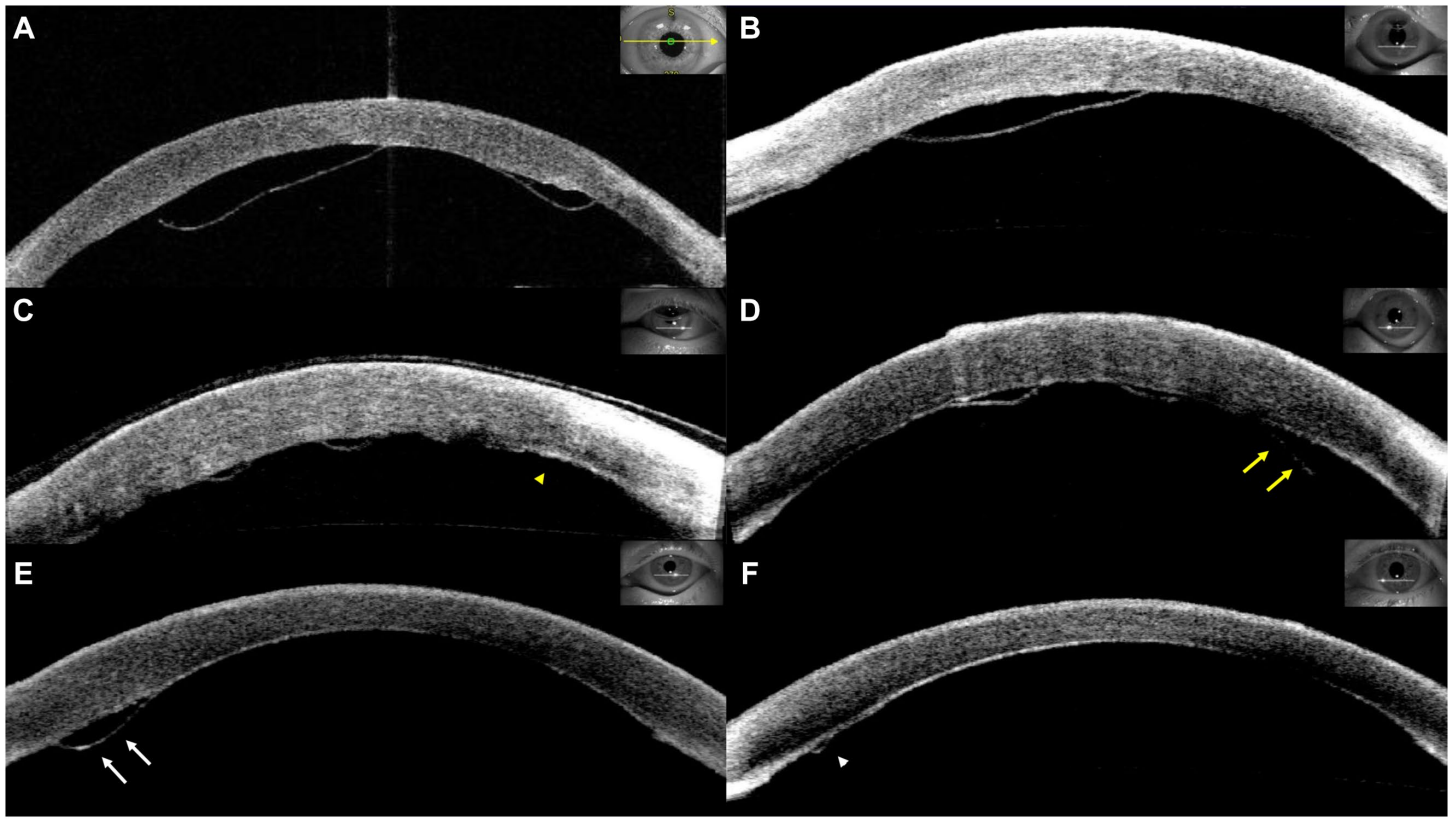
Representative cases of graft detachment. **(A)** Major detachment. **(B)** Central graft detachment within the visual axis. Progressive detachment was observed from postoperative day 1 (**C**, yellow arrowhead) to postoperative day 8 (**D**, yellow arrows). **(D)** Minor detachment was detected on postoperative day 4 (**E**, white arrows) and subsequent resolution (**F**, white arrowhead).

Graft detachment was defined as non-adherence of any portion of the graft observed through slit-lamp examination or anterior segment optical coherence tomography (AS-OCT). The degree of graft detachment was classified as minor (<1/3 of the graft surface area) or major (≥1/3 of the graft surface area). Patients with minor or major detachments were evaluated more frequently at the discretion of the surgeon. Rebubbling procedures were performed in cases of major detachment, central graft detachment along the visual axis, or progressive detachment, starting with peripheral minor detachment (Figure 1). AS-OCT scans were obtained from at least four meridians (45, 90, 135, and 180°) and evaluated by two masked observers before and after the rebubbling procedure.

Rebubbling procedures were performed in the operating room under topical and sub-Tenon anesthesia. The paracentesis site was identified and opened using forceps. A slight release of aqueous humor from the anterior chamber was achieved, followed by the injection of filtered air to achieve a complete fill (≥90%). Patients were instructed to maintain a supine position with intermittent breaks for meals and bathroom use. All rebubbling procedures were performed and recorded by an experienced DMEK surgeon (MKK). Postoperative medications included topical antibiotics and steroids, similar to those used for primary DMEK.

### Data collection

Various preoperative baseline demographic factors were assessed, including age, sex, intraocular pressure, central corneal thickness (CCT), surgical indications, preoperative lens status, and follow-up period. CCT was determined using anterior-segment optical coherence tomography (Heidelberg Engineering, Heidelberg, Germany).

Donor factors, such as age, corneal diameter, death-to-tissue preparation interval, tissue preparation-to-operation interval, and death-to-operation interval were recorded. Donor information, such as age, sex, and corneal parameters, was provided by the Eversight Eye Bank.

Recipient factors included age, sex, surgical indication (FED or BK), corneal diameter (white-to-white measured using Pentacam, Oculus, Wetzlar, Germany), axial length (measured using IOL Master 700, Carl Zeiss, Jena, Germany), CCT (measured using AS-OCT), and the severity of preoperative corneal edema, calculated as (preoperative CT-postoperative 1-month CT) divided by postoperative 1-month CT, with CT measured by AS-OCT.

Surgical factors included graft size, descemetorhexis size, graft–descemetorhexis size difference, graft–host cornea size difference, graft decentration, and postoperative 2-h IOP. Decentration of the graft was defined as stromal gaping between the descematorhexis edge and the donor graft of 1.5 mm or more over at least 3 clock hours (21). Stromal gaping was identified through postoperative anterior segment photography and AS-OCT. Postoperative 2-h IOP was measured using a handheld tonometer (iCare Finland Oy, Vantaa, Finland) in the supine position.

### Statistical analysis

Eyes were divided into two groups based on the presence or absence of graft detachment. Donor, recipient, and surgical factors were compared between the two groups. For continuous variables such as age and IOP, Student’s unpaired *t*-test was used if data were normally distributed. Normal distribution was tested using the Shapiro–Wilk test. If data were not normally distributed, the Mann–Whitney U test was used. Categorical variables were compared using the chi-square test or Fisher’s exact test, depending on the sample size and data distribution. Univariate and multivariate logistic regression analyses were performed to identify factors associated with graft detachment. Factors with a *p* value <0.1 in the univariate analysis were initially selected for a multivariate regression analysis. To meet the requirement for an overall sample size criteria (22), five variables were finally included based on *p* value (0.001–0.063) in a multivariate regression analysis. This analysis helped to determine the relationship between the occurrence of graft detachment and various factors, considering their respective odds ratios (OR) and 95% confidence intervals (CI). Statistical significance was defined as *p* < 0.05. Statistical analyses were conducted using SPSS Statistics software (version 27.0; IBM, Armonk, NY, United States).

## Results

### Demographic characteristics

The baseline characteristics of patients are presented in Table 1. A total of 52 eyes from 48 patients were included, with an average age of 65.8 ± 11.4 years. Fifty percent of these patients were male, and 50% were female. The baseline IOP was 13.9 ± 6.84 mmHg, and CCT was 729 ± 100 μm. The surgical indications included FED (36.5%), BK due to pseudophakic bullous keratopathy (27%), angle-closure glaucoma (11.6%), uveitis (11.6%), toxic anterior segment syndrome (3.8%), viral endotheliitis (3.8%), previous graft failure (3.8%), and iridocorneal endothelial (ICE) syndrome (1.9%). Cataract surgery was performed before DMEK in 96% of eyes, whereas simultaneous cataract surgery and DMEK were performed in 4%. Three patients had a history of pars planar vitrectomy due to epiretinal membrane removal. The mean duration of follow-up was 25.2 months. Graft detachment requiring rebubbling was identified in 17 eyes (32.7%) at 5 ± 2.1 days (1–8 days) postoperative.

**Table 1:** Baseline characteristics.

	Mean ± SD or *N* (%)
Age, years	65.8 ± 11.4
Sex, male/female	24/24 (50/50)
Baseline IOP, mmHg	13.9 ± 6.84
Baseline CCT, μm	729 ± 100
Surgical indication	
FED	19 (36.5)
BK	
PBK	14 (27.0)
ACG	6 (11.6)
Uveitis	6 (11.6)
TASS	2 (3.8)
Viral endotheliitis	2 (3.8)
Graft failure	2 (3.8)
ICE syndrome	1 (1.9)
Lens status	
Phakia	2 (4)
Pseudophakia	50 (96)
Underlying disease	
Diabetes	8 (16.7)
Hypertension	11 (22.9)
Sjögren’s syndrome	1 (1.9)
Juvenile rheumatoid arthritis	1 (1.9)
Follow-up, months	25.2 ± 19.1
Graft detachment	17 (32.7)

IOP, Intraocular pressure; CCT, Central corneal thickness; FED, Fuchs’ endothelial dystrophy; BK, Bullous keratopathy; PBK, Pseudophakic bullous keratopathy; ACG, Angle-closure glaucoma; TASS, Toxic anterior segment syndrome; ICE, Iridocorneal endothelial; and SD, Standard deviation.

### Donor, recipient, and surgical factors

A comparison of the donor, recipient, and surgical factors between the groups is presented in Table 2.

**Table 2:** Comparison of donor, recipient, and surgical factors between groups.

	Detachment (+) *n* = 17	Detachment (−) *n* = 35	*p* value
Donor factors			
Age, years	57.6 ± 5.81	557.5 ± 5.73 5	0.724
Corneal diameter, mm	11.2 ± 0.39	11.2 ± 0.36	0.948
Death-to-tissue prep, hours	60.6 ± 10.4	56.7 ± 14.2	0.373
Tissue prep-to-op, hours	76.8 ± 22.0	69.4 ± 17.3	0.223
Death-to-op, hours	135 ± 18.8	126 ± 13.3	0.055
Recipient factors			
Age, years	71.7 ± 8.96	63.1 ± 11.5	**0.006**
Sex, male/female	7/7	17/17	0.999
Diabetes	7	1	**0.001**
Surgical indication, FED/BK	11/6	8/27	**0.003**
Corneal diameter, mm	11.1 ± 0.58	10.9 ± 0.62	0.565
Axial length, mm	24.9 ± 2.97	24.0 ± 2.75	0.238
Central corneal thickness, μm	735 ± 96.3	726 ± 103	0.739
Severity of preop corneal edema*	40.0 ± 24.6	40.6 ± 19.9	0.953
Surgical factors			
Graft size, mm	7.75 ± 0.32	7.71 ± 0.41	0.500
Descemetorhexis size, mm	8.44 ± 0.43	8.38 ± 0.47	0.605
Graft–descemetorhexis size difference, mm	0.69 ± 0.33	0.67 ± 0.39	0.508
Graft–host cornea size difference, mm	3.32 ± 0.49	3.22 ± 0.68	0.430
Endothelium-in/out	14/3	24/11	0.293
Decentration of graft	6	5	0.145
Postop 2-h IOP, mmHg	13.6 ± 4.26	22.2 ± 9.54	**0.002**
Postop 2-h IOP, IOP <20 mmHg/≥20 mmHg	16/1	16/19	**<0.001**

FED, Fuchs’ endothelial dystrophy; BK, Bullous keratopathy; CT, Corneal thickness; and IOP, Intraocular pressure.

^*^Calculated as (preop CT-postop 1-month CT)/postop 1-month CT, %.

Values with statistical significance are shown in boldface.

Among all donor factors, no statistically significant differences were observed between the two groups. However, the death-to-operation time showed a marginal significance, with a longer duration observed in the detachment group (135 ± 18.8 h) compared with the non-detachment group (126 ± 13.3 h) (*p* = 0.055).

Among the eight recipient factors, sex, axial length, and corneal dimensions were not significantly different between the two groups. The level of corneal edema, as indicated by CCT, and the severity of preoperative corneal edema also did not differ between groups. However, recipient age, diabetes and surgical indications displayed notable differences. The detachment group had a higher mean recipient age (71.7 ± 8.96 years) compared with the non-detachment group (63.1 ± 11.5 years) (*p* = 0.006). Prevalence of diabetes was higher in detachment group than non-detachment group (*p* = 0.001). Additionally, the proportion of patients with FED as a surgical indication was significantly higher in the detachment group (65%) than in the non-detachment group (23%) (*p* = 0.003).

We investigated various surgical factors. Graft and descemetorhexis sizes did not differ significantly between the two groups. Similarly, the size difference between the DMEK graft and the descemetorhexis or host cornea was not significant. The handling of grafts as endothelium-in or endothelium-out did not result in any differences. The presence of graft decentration was identified in six eyes in the detachment group and five eyes in the non-detachment group (*p* = 0.145). However, a clear difference was observed in the postoperative 2-h IOP. The detachment group exhibited a significantly lower postoperative 2-h IOP (13.6 ± 4.26 mmHg) compared with the non-detachment group (22.2 ± 9.54 mmHg) (*p* = 0.002). When categorized as lower or higher than 20 mmHg, a higher proportion of eyes with IOP lower than 20 mmHg was observed in the detachment group (94%) compared with the non-detachment group (46%) (*p* < 0.001).

### Factors associated with graft detachment

Logistic regression analyses were conducted to identify risk factors associated with graft detachment requiring rebubbling, and the analyzed factors and odds ratios are presented in Table 3. In the univariate analysis, older recipient age (OR 1.08, 95% CI 1.02–1.17, *p* = 0.016), diabetes (OR 23.8, 95% CI 2.61–217, *p* = 0.001), a surgical indication of FED (OR 6.19, 95% CI 1.74–22.0, *p* = 0.005), lower postoperative 2-h IOP (OR 1.21, 95% CI 1.06–1.38, *p* = 0.005), and postoperative 2-h IOP <20 mmHg (OR 14.0, 95% CI 1.64–119, *p* = 0.016) were identified as factors increasing the odds of graft detachment.

**Table 3:** Factors associated with graft detachment requiring rebubbling (five variables for multivariate logistic regression).

	Univariate		Multivariate	
	OR (95% CI)	*p* value	OR (95% CI)	*p* value
Donor factors				
Age, years	1.00 (0.91–1.11)	0.937	NA	NA
Death-to-tissue prep, hours	1.02 (0.97–1.07)	0.366	NA	NA
Tissue prep-to-op, hours	1.02 (0.98–1.06)	0.222	NA	NA
Death-to-op, hours	1.04 (0.99–1.09)	0.063	1.06 (0.99–1.13)†	0.111†
			1.05 (0.98–1.12)‡	0.160‡
Recipient factors				
Age, years	**1.08** (1.02–1.17)	**0.016**	1.00 (0.90–1.12)†	0.972†
			1.01 (0.91–1.11)‡	0.854‡
Diabetes	**23.8** (2.61–217)	**0.001**	**23.6** (1.28–400)†	**0.034**†
			**25.3** (1.89–460)‡	**0.029**‡
Surgical indication, FED	**6.19** (1.74–22.0)	**0.005**	4.33 (0.67–27.9)†	0.123†
			5.41 (0.88–33.1)‡	0.068‡
Corneal diameter, mm	1.15 (0.54–3.85)	0.458	NA	NA
Axial length, mm	1.11 (0.91–1.36)	0.292	NA	NA
Central corneal thickness, μm	1.00 (0.99–1.01)	0.740	NA	NA
Severity of preop corneal edema*, %	0.99 (0.97–1.02)	0.929	NA	NA
Surgical factors				
Graft size, mm	1.27 (0.28–5.78)	0.751	NA	NA
Descemetorhexis size, mm	1.31 (0.36–4.73)	0.677	NA	NA
Graft–descemetorhexis size difference, mm	1.16 (0.24–5.52)	0.856	NA	NA
Graft–host cornea size difference, mm	1.29 (0.50–3.33)	0.594	NA	NA
Decentration of graft	3.27 (0.83–12.9)	0.091	NA	NA
Postop 2-h IOP, mmHg	**1.21** (1.06–1.38)	**0.005**	**1.23** (1.02–1.47)†	**0.027**†
Postop 2-h IOP, IOP <20 mmHg	**14.0** (1.64–119)	**0.016**	**25.1** (1.05–602)‡	**0.047**‡

OR, odds ratio; CI, confidence interval; NA, not available; FED, Fuchs’ endothelial dystrophy; IOP, intraocular pressure; and CT, corneal thickness.

^*^Calculated as (preop CT-postop 1-month CT)/postop 1-month CT, %.

^†^Multivariate logistic regression model including death-to-op hours, recipient age, diabetes, surgical indication, and postop 2-h IOP (continuous value) as parameters.

^‡^Multivariate logistic regression model including death-to-op hours, recipient age, diabetes, surgical indication, and postop 2-h IOP < 20 mmHg (categorical value) as parameters.

Values with statistical significance are shown in boldface.

**Figure 2:**
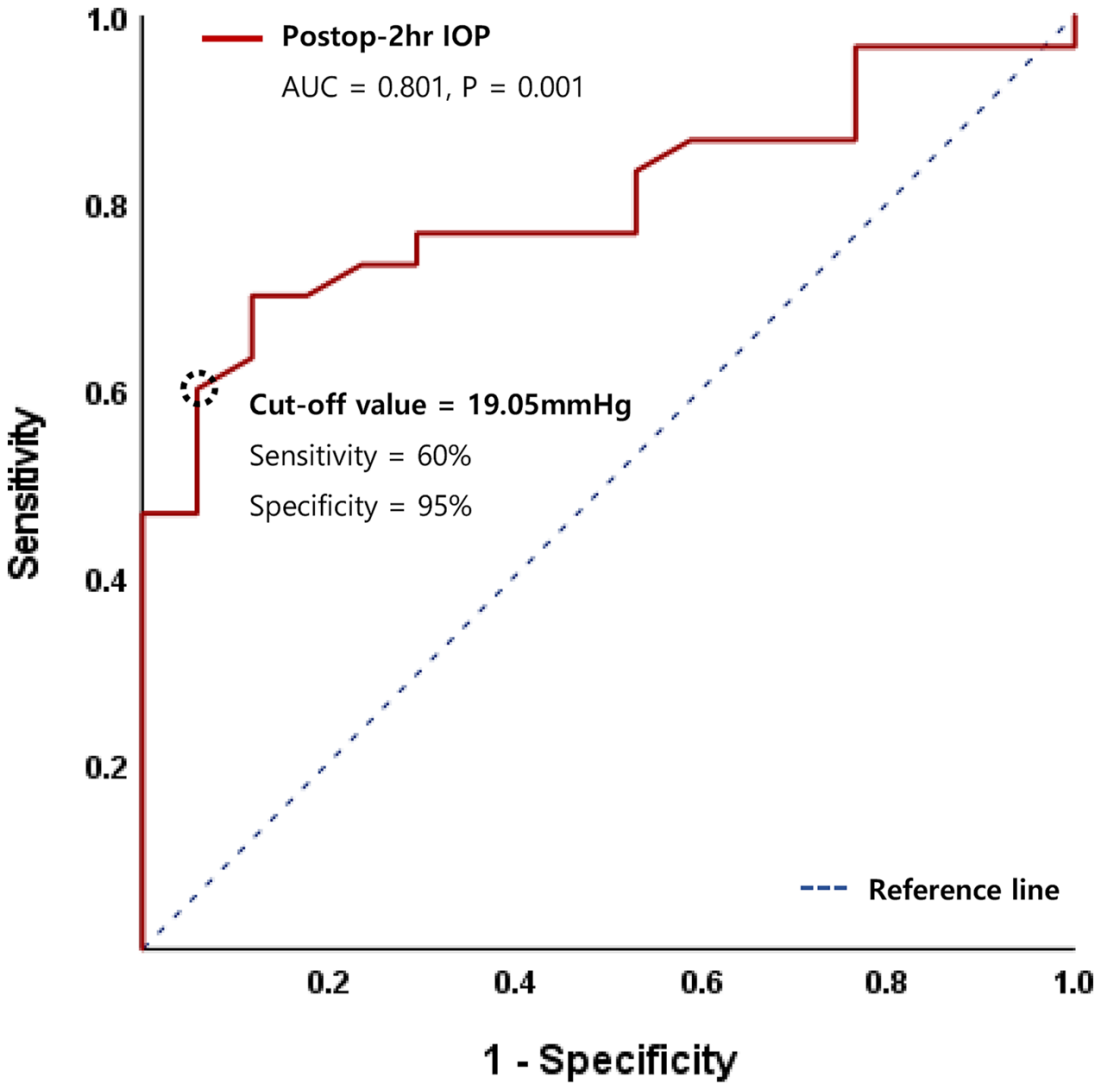
Receiver operating characteristic (ROC) curve for logistic regression model of postoperative 2-h intraocular pressure (IOP).

In multivariate logistic regression analysis, recipient age and surgical indication of FED no longer remained significant. However, diabetes (OR 23.6, 95% CI 1.28–400, *p* = 0.034 or OR 25.3, 95% CI 1.89–460, *p* = 0.029), lower postoperative 2-h IOP (OR 1.23, 95% CI 1.02–1.47, *p* = 0.027), and postoperative 2-h IOP <20 mmHg (OR 25.1, 95% CI 1.05–602, *p* = 0.047) remained significant graft detachment risk factors. Additionally, a receiver operating characteristic (ROC) curve was generated, and the area under the curve (AUC) value was analyzed to identify the cut-off IOP value predicting graft detachment. The AUC for postoperative 2-h IOP was 0.801 (*p* = 0.001), with a cut-off IOP of 19.05 mmHg, demonstrating 60% sensitivity and 95% specificity (Figure 2).

### Visual recovery and endothelial cell density change

**Figure 3:**
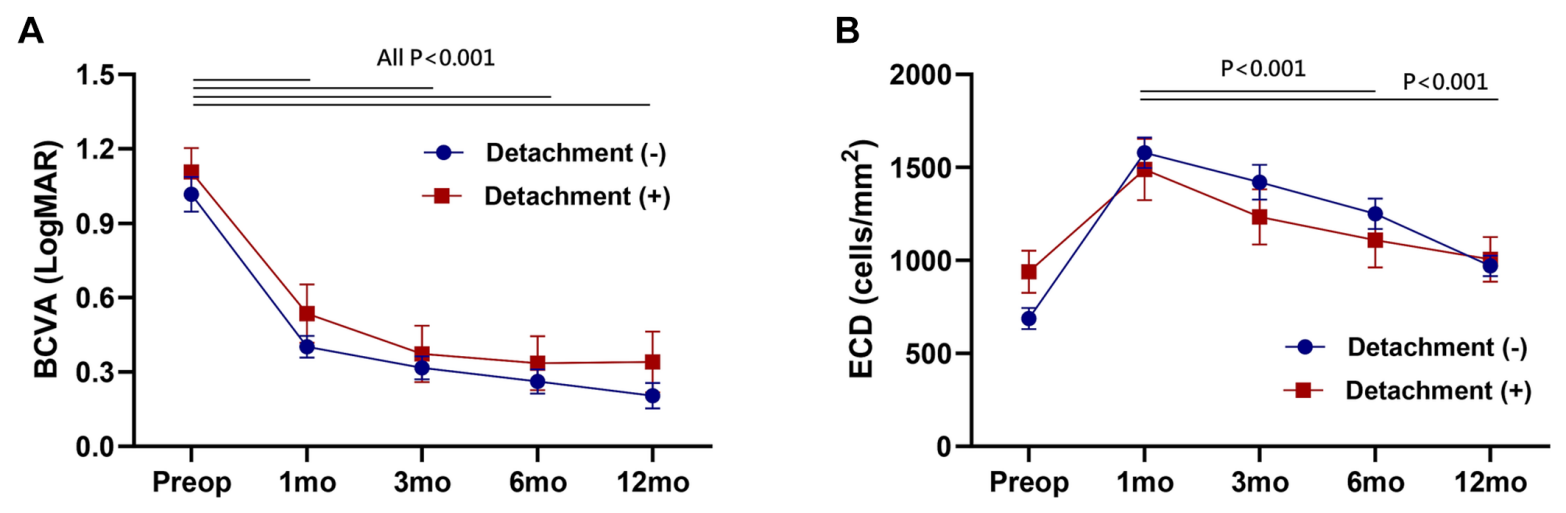
Postoperative change of best-corrected visual acuity (BCVA) **(A)** and endothelial cell density (ECD) **(B)**. No significant difference between the detachment and non-detachment group at each follow-up period (Unpaired *t*-test, *p* > 0.05). Data are presented as the mean ± standard error of the mean.

In intra-group analysis, best-corrected visual acuity (BCVA) demonstrated a significant improvement following DMEK throughout all postoperative follow-up periods (All *p* < 0.001). Endothelial cell density (ECD) exhibited a continuous decrease over 12 months, and this decline was statistically significant (Paired *t*-test, 1 month vs. 6 and 12 months, *p* < 0.001, respectively). In inter-group analysis, however, no significant differences were observed between the detachment group and the non-detachment group in each follow-up period (Figure 3).

## Discussion

This study observed an overall graft detachment rate of 32.7%, which is consistent with that reported by previous studies (5). Diabetes exhibited a higher risk of graft detachment. Additionally, a lower postoperative 2-h IOP was associated with an increased graft detachment risk. Notably, a postoperative 2-h IOP lower than 20 mmHg was a significant risk factor, underscoring the importance of maintaining immediate postoperative IOP levels above 20 mmHg to prevent graft detachment.

Several studies have explored the role of postoperative IOP in graft detachment after DMEK. Schmeckenbächer et al. (12) have reported no association between the initial IOP magnitude and incomplete graft adhesion in DMEK. Also, no evidence suggested that higher IOP promotes graft adhesion leading to a lower rebubbling rate. In the same year, Pilger et al. (23) have reported that maintaining an IOP within the range of 10–20 mmHg with postsurgical air tamponade for at least 2 h reduces the rebubbling rate in DMEK. Furthermore, while Heinzelmann et al. (24) have reported that the first or maximum IOP does not influence graft detachment, relative dips in IOP are associated with a higher risk. However, in our investigation, IOPs below 20 mmHg were found to be associated with an increased risk of graft detachment. Analysis of the ROC curve indicated a cut-off IOP value predicting graft detachment at 19.05 mmHg with a high specificity of 95% and sensitivity of 60%. This is a clinically important finding in that maintaining IOP higher than the cut-off value could prevent graft detachment. It is also noteworthy that this is the first report identifying a cut-off IOP value with regard to graft detachment. Our findings suggest that meticulous closure of surgical wounds and optimal filling of the anterior chamber are crucial for maintaining an adequate IOP level, thereby preventing graft detachment. Interestingly, our data contradicted a prior report by Pilger et al. (22) which demonstrated increased graft detachment and rebubbling in eyes with an IOP higher than 20 mmHg. Further studies, incorporating larger sample sizes and prospective designs, are warranted to reconcile these discrepancies.

Diabetes emerged as a significant contributor to the risk of graft detachment in our study. Price et al. (25) reported on the impact of donor and recipient diabetes status on DMEK graft adherence and survival. While donor diabetes was not associated with air reinjection, graft survival, or endothelial cell loss, recipient diabetes correlated with increased endothelial cell loss. Varied Descemet membrane adhesion strength (26) and cleavage plane alterations after stripping the recipient Descemet membrane (27) have been reported in diabetic recipients, collectively influencing DMEK graft adherence. In our study, diabetes exhibited the highest odds of graft detachment (OR 23.6–33.6). Building upon previous reports, this study reinforces the significance of host diabetes in influencing the postoperative graft adherence.

The proportion of cases with FED was notably higher in the graft detachment group, with FED demonstrating a significantly high OR in univariate regression analysis. Given the increased prevalence of both FED and diabetes in the detachment group compared to the non-detachment group, a potential confounding effect between these variables was suspected. Cross-analysis revealed a significant association between diabetes and FED in the detachment group (Fisher’s exact test, *p* = 0.001). Consequently, FED lost significance in multivariate analysis.

Although longer graft storage time (13) and lower graft ECD (14) may influence graft detachment and placement rates, these factors did not significantly differ between the detachment and non-detachment groups. Further analysis is needed to explore the association between tissue preservation time and ECD, along with their relationship to graft attachment. Despite achieving an overall graft detachment rate comparable to that reported in previous studies (32.7%), room for improvement remains. One factor that may have contributed to our findings is the scarcity of corneal donors in Korea, which necessitates reliance on imported pre-cut tissues. Unlike in Western countries where pre-cut tissue is readily available within 24–48 h, the longer death-to-operation time observed in our study group can be attributed to this limitation. Although the significance between these two groups was only marginally significant, a previous study has reported an association between graft storage time and adherence (13). Therefore, shortening the tissue-preservation time may contribute to reducing the graft detachment rate in our setting.

Given that the average time interval for graft detachment was 5 ± 2.1 days (ranging from 1 to 8 days), it appears that most graft detachments occurred early in the postoperative period. Although we did not explore the differences in postoperative outcomes based on the detachment time, our analysis revealed that the presence of detachment requiring rebubbling did not result in any statistically significant differences in both visual outcomes and endothelial cell density up to 1 year postoperatively. However, in light of a recent study suggesting that rebubbling may impact endothelial cell density (28), further evaluation is warranted to assess the effects of early graft detachment on long-term outcomes.

This study has several limitations. First, the retrospective design introduced the possibility of missing data, which may have limited the accuracy and generalizability of the findings. Second, the number of cases in the graft detachment group was relatively small compared with that in the non-detachment group. Initially, we included six variables with a *p* value less than 0.1 in the multivariate regression analysis (Supplementary Table S1). However, the sample size (52 cases) did not meet the minimum requirements for such an analysis. Conventionally, for multivariate data analysis, such as regression analysis, the sample size should ideally be 10 times greater than the number of variables included (22). In consequence, we ultimately included five variables to meet overall sample size criteria (Table 3). Despite this adjustment, there was no deviation in terms of the identified significant risk factors. While the analysis demonstrated statistical significance, additional case recruitment is necessary to augment statistical power and fortify the robustness of the findings. Third, the rarity of FED in Asia poses a challenge due to the diversity of surgical indications in the current study. This limits our ability to generalize or directly compare our findings with reports from Western countries with higher FED prevalence. Nevertheless, our study serves as a valuable reference for future investigations that encompass a wider range of surgical indications, thereby facilitating the design and execution of comparative analyses. Overall, although this study has limitations, meaningful insights are provided into graft detachment risk factors and considerations in DMEK. Further studies with larger sample sizes, prospective designs, and diverse representations of surgical indications are required to broaden the understanding of this topic.

In conclusion, recipient diabetes poses a higher graft detachment risk in DMEK. To minimize graft detachment occurrence and the need for subsequent rebubbling procedures, low postoperative IOP should be avoided, and IOP levels should be maintained above 20 mmHg.

## Data availability statement

The original contributions presented in the study are included in the article/Supplementary material; further inquiries can be directed to the corresponding author.

## Ethics statement

The studies involving humans were approved by Seoul National University Institutional Review Board. The studies were conducted in accordance with the local legislation and institutional requirements. The ethics committee/institutional review board waived the requirement of written informed consent for participation from the participants or the participants’ legal guardians/next of kin because due to the retrospective nature of the study.

## Author contributions

CK: Investigation, Writing – original draft, Writing – review & editing. CY: Investigation, Writing – review & editing. MK: Conceptualization, Investigation, Writing – review & editing.
