# Supplementary material for: Risk factors for early graft detachment requiring rebubbling in Descemet membrane endothelial keratoplasty with imported pre-cut donor tissues
Source: Front Med (Lausanne). 2024 Feb 8;11:1266049. doi: 10.3389/fmed.2024.1266049 (PMC10881667; doi:10.3389/fmed.2024.1266049)
Supplement: Supplementary file 1 [file Data_Sheet_1.docx]

| Supplementary Table S1. Factors associated with graft detachment requiring rebubbling (6 variables for multivariate logistic regression) | | | | | |
| --- | --- | --- | --- | --- | --- |
|  | Univariate | | | Multivariate | |
|  | | OR (95% CI) | P-value | OR (95% CI) | P-value |
| Donor factors | |  |  |  |  |
| Age, years | | 1.00 (0.91–1.11) | 0.937 | NA | NA |
| Death-to-tissue prep, hours | | 1.02 (0.97–1.07) | 0.366 | NA | NA |
| Tissue prep-to-op, hours | | 1.02 (0.98–1.06) | 0.222 | NA | NA |
| Death-to-op, hours | | 1.04 (0.99–1.09) | 0.063 | 1.05 (0.99–1.12)†  1.05 (0.98–1.12)‡ | 0.131†  0.146‡ |
| Recipient factors | |  |  |  |  |
| Age, years | | **1.08** (1.02–1.17) | **0.016** | 1.01 (0.90–1.12)†  1.02 (0.92–1.13)‡ | 0.918†  0.728‡ |
| Diabetes | | **23.8** (2.61-217) | **0.001** | **23.6** (1.28–400)†  **33.6** (1.89–597)‡ | **0.034**†  **0.017**‡ |
| Surgical indication, FED | | **6.19** (1.74–22.0) | **0.005** | 4.23 (0.61–29.3)†  5.46 (0.88–33.8)‡ | 0.145†  0.068‡ |
| Corneal diameter, mm | | 1.15 (0.54–3.85) | 0.458 | NA | NA |
| Axial length, mm | | 1.11 (0.91–1.36) | 0.292 | NA | NA |
| Central corneal thickness, µm | | 1.00 (0.99–1.01) | 0.740 | NA | NA |
| Severity of preop corneal edema*, % | | 0.99 (0.97-1.02) | 0.929 | NA | NA |
| Surgical factors | |  |  |  |  |
| Graft size, mm | | 1.27 (0.28–5.78) | 0.751 | NA | NA |
| Descemetorhexis size, mm | | 1.31 (0.36–4.73) | 0.677 | NA | NA |
| Graft–descemetorhexis size difference, mm | | 1.16 (0.24–5.52) | 0.856 | NA | NA |
| Graft–host cornea size difference, mm | | 1.29 (0.50–3.33) | 0.594 | NA | NA |
| Decentration of graft | | 3.27 (0.83–12.9) | 0.091 | 3.45 (0.38–30.8)†  3.06 (0.40–23.4)‡ | 0.267†  0.281‡ |
| Postop 2-h IOP, mmHg | | **1.21** (1.06–1.38) | **0.005** | **1.24** (1.03–1.50)† | **0.026**† |
| Postop 2-h IOP, IOP <20 mmHg | | **14.0** (1.64–119) | **0.016** | **21.3** (0.86–532)‡ | **0.050**‡ |
| OR, odds ratio; CI, confidence interval; NA, not available; FED, Fuchs’ endothelial dystrophy; IOP, intraocular pressure; CT, corneal thickness.  *Calculated as (preop CT-postop one-month CT)/postop one-month CT, %  †Multivariate logistic regression model including death-to-op hours, recipient age, diabetes, surgical indication, decentration of graft, postop 2-h IOP (continuous value) as parameters.  ‡Multivariate logistic regression model including death-to-op hours, recipient age, diabetes, surgical indication, decentration of graft, postop 2-h IOP<20 mmHg (categorical value) as parameters.  Values with statistical significance are shown in boldface. | | | | | |
